# Results of new-generation balloon vs. self-expandable transcatheter heart valves for bicuspid aortic valve stenosis

**DOI:** 10.3389/fcvm.2023.1252163

**Published:** 2023-09-01

**Authors:** Oliver Deutsch, Keti Vitanova, Hendrik Ruge, Magdalena Erlebach, Markus Krane, Rüdiger Lange

**Affiliations:** ^1^ Department of Cardiovascular Surgery, German Heart Centre Munich, Munich, Germany; ^2^ Department of Surgery, Division of Cardiac Surgery, Yale School of Medicine, New Haven, CT, United States

**Keywords:** TAVR - transcatheter aortic valve replacement, bicuspid aortic valve (BAV), balloon-expandable valve, self-expandable valve, retrospective cohort study

## Abstract

**Background:**

Data comparing new-generation self-expandable (SEV, Evolut R/PRO) vs. balloon-expandable (BEV, SAPIEN 3/3Ultra) transcatheter heart valve replacement (TAVR) in bicuspid aortic valve stenosis (BAV) is limited. Our aim was to compare 30-day results of SEV and BEV implantations in patients with BAV.

**Methods:**

A total of 2009 patients underwent TAVR between April 2015 and June 2021 at our Centre. From our institutional registry, we identified 106 consecutive patients with BAV who underwent TAVR using SEV and BEV.

**Results:**

A 106 patients (*n* = 68 BEV; *n* = 38 SEV) were included. Mean age was 74.6 ± 8.8 years (BEV) vs.75.3 ± 8.7 years (SEV) (*p* = 0.670) and Society of Thoracic Surgeons score was 2.6 ± 1.9 (BEV) vs. 2.6 ± 1.6 (SEV) (*p* = 0.374), respectively. Device landing zone calcium volume (DLZ-CV) was 1168 ± 811 vs. 945 ± 850 mm^3^ (*p* = 0.192). Valve Academic Research Consortium (VARC)-3 device success at 30 days was similar (BEV 80.9% vs. SEV 86.8%; *p* = 0.433). More post-dilatations were performed in SEVs (23.5% BEV vs. 52.6% SEV; *p* = 0.002). Overall mean gradient at 30 days follow-up was 11.9 ± 4.6 mmHG (BEV) vs. 9.2 ± 3.0 mmHG (SEV) (*p* = 0.002). A mild-moderate degree of paravalvular leak (PVL) was detected more often in the SEV group (7.4% vs. 13.2%; *p* = 0.305). A trend towards higher rate of permanent pacemaker implantation was observed in SEV (11.8% vs. 23.7%; *p* = 0.109).

**Conclusions:**

Treatment of BAV revealed similar performance using BEV and SEV. In this retrospective cohort study, hemodynamics were more favorable with the SEV, although there was a trend toward more PVL and significantly more post-dilations.

## Introduction

Bicuspid aortic valve (BAV) is the most frequent inherited valvular defect with an estimated prevalence of 0.5%–2.0% in the general population (1, 2). Currently, percutaneous treatment options are not only reserved for high risk and elderly patients but are increasingly offered to a younger population in which the prevalence of BAV is higher compared to elderly patients (1, 3–6). Compared with tricuspid aortic valves (TAV), patients with BAV are considered to be more challenging to treat with transcatheter aortic valve replacement (TAVR) due to specific anatomic factors, e.g., larger aortic annulus dimensions, long valve leaflets, a pronounced ovality of the aortic valve orifice and severe asymmetric calcification (6–8). Therefore, patients with BAV undergoing TAVR might be at higher risk of paravalvular regurgitation, annular rupture, aortic dissection, device under-expansion, and need for a second transcatheter heart valve (THV) (9, 10). So far, BAV has been an exclusion criterion in the large pivotal trials (11–15). Until recently, only results from first and second-generation balloon- or self-expanding valves were available (16–19).

Newer generation THVs have been shown to achieve higher devices success rates compared to early generation devices, encouraging a more frequent application of TAVR in BAV anatomy. The Society of Thoracic Surgeons/American College of Cardiology Transcatheter Valve Therapy (TVT) Registry reports comparable 30-day and 1-year mortality and 30-day and 1-year stroke rates for patients with BAV undergoing TAVR with third-generation self-expanding THV (19, 20).

Few data have been reported for the current generation BEV and SEV in BAV using the 2021 Valve Academic Research Consortium (VARC) defined outcome parameters for standardized reporting of VARC-3 endpoints (21–24). We aimed to compare the 30-day VARC-3 safety and efficacy outcomes of 106 consecutive patients within the investigation period with bicuspid aortic valve anatomy undergoing TAVR with the latest generation balloon-expandable (BEV, SAPIEN 3/3Ultra (Edwards Lifesciences Corp., Irvine, CA, USA) and self-expandable (SEV, Evolut R/PRO (Medtronic PLC, Dublin, Ireland) THV.

## Methods

### Study population

Among 2009 consecutive patients with native aortic valve disease treated at the Department of Cardiovascular Surgery of the German Heart Centre in Munich, Germany, between April 2015 and June 2021, we identified 123 patients with biscuspid valve morphology who underwent TAVR. Pre-operative clinical data, procedural and post-operative outcomes with at least 30-days of follow-up were analyzed. Based on contrast-enhanced multi-detector CT (MDCT) reconstruction of the aortic valve, patients were categorized as BAV anatomy, in accordance with standard definitions (18, 23). In cases of concomitant coronary artery disease BEV was preferred in order to enable optimal access for future coronary interventions. Nine cases were excluded because devices used were other than the specified THVs and two international patients were excluded due to lack of follow-up. Six cases of BEV implantation via trans-apical access were excluded due to predetermined valve selection. Embolic protection devices were not used in this study. A Heart Team consisting of cardiologists and cardiac surgeons performed an evaluation of surgical risk in every case: patients were suitable for TAVR if the surgical risk was regarded to be intermediate or high, based on the logistic EuroSCORE and Society of Thoracic Surgeons (STS) predicted risk of mortality (PROM) score and individual clinical factors not represented by traditional risk scores. The study was conducted in accordance with the Declaration of Helsinki (as revised in 2013). The study was approved by institutional ethics committee (Technical University Munich, Faculty of Medicine, ethics committee vote number 487/21 S) and informed consent was waived for this retrospective study.

### Multi-detector computed tomography

All patients underwent contrast-enhanced MDCT of the aortic root and access vessels prior to TAVR. All datasets were analyzed offline using FDA-approved software (3mensio, Pie Medical Imaging BV, Maastricht, The Netherlands). Aortic annulus sizing was executed according to tricuspid sizing techniques by measuring minimal and maximal diameter, area, and perimeter. Currently recommended sizing algorhithms were stepwise adopted as sizing strategies were emerging during the study period (25, 26). This applies also for sizing of Sievers type 0 and type 1 morphologies (23). The calcification pattern was categorized in none/minimal, mild, mild-moderate, moderate, moderate-severe, and severe. Calcium volume measurements were performed using contrast-enhanced MDCT scans with the previously described approximation method (24). Device landing zone calcium volume (DLZ-CV) was measured including the aortic valve at the top of the commissures and the left ventricular outflow tract 5 mm below the annular plane. We used a validated contrast-enhanced scan-specific threshold (Mean Hounsfield units + 4 × standard deviation), determined by measuring directly above the aortic valve in the aortic root, with a 5 mm^3^ volume filter (27).

### Transthoracic echocardiography

Transthoracic echocardiography (TTE) was performed at baseline, pre-discharge, and during the follow-up period of at least 30 days. TTE parameters were collected by conventional M-mode, Doppler and two-dimensional assessment. Left ventricular ejection fraction (LVEF) was measured using the Simpson method in the two and four-chamber apical 2D view. Valve area was determined on the basis of the continuity equation. Mean transvalvular gradient and maximum velocity were collected. PVL were evaluated after the procedure by using an integrative approach, according to the American Society of Echocardiography guidelines, and then categorized as none/trace, mild, moderate, and severe (28). See Table 1 “Baseline characteristics”.

**Table 1:** Baseline characteristics.

	Overall (*n* = 106)	BEV (*n* = 68)	SEV (*n* = 38)	*p*-value
Age, year		74.6 ± 8.8	75.3 ± 8.7	0.670
Male sex	69/106 (65%)	49/68 (72.1%)	20/38 (52.6%)	0.044
BMI		28.8 ± 11.3	25.6 ± 5.2	0.100
Pulmonary disease	10/106 (9.4%)	7/68 (10.3%)	3/38 (7.9%)	0.685
Serum creatinine, mg/dl		1.3 ± 1.2	1.1 ± 0.4	0.620
Coronary artery disease	45/106 (42.5%)	33/68 (48.5%)	12/38 (31.6%)	0.090
Natriuretic peptide, ng/ml		3,785 ± 6,492	3,113 ± 3,497	0.625
Prior cardiac surgery	6/106 (5.7%)	4/68 (5.9%)	2/38 (5.3%)	0.895
Cerebrovascular disease	5/106 (4.7%)	5/68 (7.4%)	0/38 (0%)	0.083
Prior TIA or stroke	5/106 (4.7%)	4/68 (5.9%)	1/38 (2.6%)	0.449
Peripheral artery disease	8/106 (7.5%)	6/68 (16.2%)	2/38 (5.3%)	0.493
Pulmonary hypertension	10/106 (9.5%)	8/68 (11.8%)	2/38 (5.3%)	0.272
Atrial fibrillation	18/106 (16.9%)	13/68 (19.1%)	5/38 (13.2%)	0.611
Prior permanent pacemaker	9/106 (8.5%)	5/68 (7.4%)	4/38 (10.5%)	0.574
STS PROM, %		2.6 ± 1.9	2.6 ± 1.6	0.374
EuroSCORE II, %		3.9 ± 3.3	2.8 ± 1.6	0.074
EuroSCORE logistic, %		11.6 ± 9.2	9.3 ± 6.3	0.165
Frailty	34/106 (32.1%)	19/68 (27.9%)	15/38 (39.5%)	0.223
NYHA class				0.046
I	0/106 (0%)	0/68 (0%)	0/38 (0%)	
II	13/106 (12.3%)	11/68 (16.2%)	2/38 (5.3%)	
III	88/106 (83%)	52/68 (76.4%)	36/38 (94.7%)	
IV	5/106 (4.7%)	5/68 (7.4%)	0/38 (0%)	
Echocardiographic data				
Mean AV gradient, mmHG		44.5 ± 15.4	48.3 ± 18.3	0.255
AVA, cm^2^		0.74 ± 0.2	0.73 ± 0.2	0.702
Moderate aortic regurgitation	4/106 (3.8%)	4/68 (5.9%)	0/38 (0%)	0.343
LVEF, %		51.2 ± 14.1	54.0 ± 12.4	0.308
MDCT data				
Area, mm^2^		536 ± 109	467 ± 114	0.003
Perimeter, mm		83.4 ± 0.9	77.8 ± 0.9	0.004
Effective diameter, mm		26.5 ± 2.7	24.7 ± 3.1	0.003
Minimum diameter, mm		23.2 ± 2.9	21.7 ± 3.1	0.019
Maximum diameter, mm		29.4 ± 3.0	27.4 ± 3.2	0.002
Device landing zone calcium volume, mm^3^		1,168 ± 811	948 ± 850	0.192
Leaflet				
NCC calcium volume, mm^3^		350 ± 287	281 ± 266	0.224
RCC calcium volume, mm^3^		263 ± 228	250 ± 283	0.807
LCC calcium volume, mm^3^		241 ± 213	210 ± 208	0.466
Annulus				
NCC calcium volume, mm^3^		97 ± 129	60 ± 82	0.107
RCC calcium volume, mm^3^		82 ± 126	64 ± 90	0.442
LCC calcium volume, mm^3^		66 ± 106	44 ± 56	0.231
LVOT				
NCC calcium volume, mm^3^		29 ± 43	23 ± 49	0.497
RCC calcium volume, mm^3^		19 ± 45	16 ± 33	0.699
LCC calcium volume, mm^3^		23 ± 55	14 ± 31	0.398
Bulky calcification	33/106 (31.1%)	22/68 (32.4%)	11/38 (28.9%)	0.446
Aortic valve calcification				0.445
None	2/106 (1.9%)	2/68 (29%)	0/38 (0%)	
Mild	22/106 (20.1%)	10/68 (14.7%)	9/38 (23.7%)	
Moderate	61/106 (57.5%)	37/68 (54.4%)	21/38 (55.3%)	
Severe	27/106 (25.5%)	19/68 (27.9%)	8/38 (21.1%)	
Type of bicuspid AV (Sievers)				0.119
Type 0	8/106 (7.5%)	6/68 (8.8%)	2/38 (5.3%)	
Type 1 L/R	87/106 (82.1%)	57/68 (83.8%)	30/38 (78.9%)	
Type 1 R/N	8/106 (7.5%)	5/68 (7.4%)	3/38 (7.9%)	
Type 1 N/L	3/106 (2.8%)	0/68 (0%)	3/38 (7.9%)	
Type 2	0/106 (0%)	0/68 (0%)	0/38 (0%)	

Values are mean ± SD or *n* (%). AV, aortic valve; AVA, aortic valve area; BMI, body mass index; EuroSCORE, European system for cardiac operative risk; LCC, left coronary cusp; LVEF, left ventricular ejection fraction; LVOT, left ventricular outflow tract; L/R, left/right; MDCT, multidetector computed tomography; NCC, non-coronary cusp; NYHA, New York heart association evaluation; N/L, non/left; PROM, predicted risk of mortality; RCC, right coronary cusp; R/N, right/non; STS, society of thoracic surgeons; TIA, transient ischemic attack.

### Transcatheter aortic valve implantation strategy

Valve selection (BEV, SAPIEN 3/3Ultra (Edwards Lifesciences Corp., Irvine, CA, USA and SEV, Evolut R/PRO (Medtronic PLC, Dublin, Ireland) was at the discretion of our local Heart Team. Valve size was chosen according to the manufacturers’ recommendations on the basis of CT-measurements. The access route was decided according to the preoperative scan's data. A transfemoral access was preferred if possible. Procedures were performed under local anaesthesia and conscious sedation or under general anaesthesia in cases with trans-aortic or transaxillary access. There was no dedicated protocol for pre-dilation of the native aortic valve and thus remained at the operator's discretion. THV function was assessed during the procedure to determine the transvalvular gradient and aortography performed to evaluate for residual aortic regurgitation (29). The temporary pacing wire was delivered via the jugular vein in most cases.

### Endpoints

Primary endpoints were selected in accordance with the Valve Academic Research Consortium (VARC)-3 criteria (21): 30-day mortality, technical success (at exit from procedure room), device success (at 30 days), and early safety (at 30 days).

Secondary endpoints included the need for permanent pacemaker implantation, as well as procedure-related variables, i.e., pre- and post-dilation or need for implant of a second valve. See Table 2 “Procedural characteristics and clinical outcomes”.

**Table 2:** Procedural characteristics and clinical outcome.

	Overall (*n* = 106)	BEV (*n* = 68)	SEV (*n* = 38)	*p*-value
Conscious sedation	71/106 (66.9%)	46/68 (67.6%)	25/38 (65.8%)	0.405
Access route				0.912
Transfemoral	101/106 (95.2%)	65/68 (95.6%)	36/38 (94.7%)	
Transubclavian	2/106 (1.9%)	1/68 (1.5%)	1/38 (2.6%)	
Direct aortic	3/106 (2.8%)	2/68 (2.9%)	1/38 (2.6%)	
Valve size				<0.001
23 mm	17/106 (16%)	14/68 (20.6%)	3/38 (7.9%)	
26 mm	29/106 (27.4%)	20/68 (29.4%)	9/38 (23.7%)	
29 mm	48/106 (45.3%)	34/68 (50%)	14/38 (36.8%)	
34 mm	12/106 (11.3%)	0/74 (0%)	12/38 (31.6%)	
Pre-dilation	57/106 (53.8%)	39/68 (57.4%)	18/38 (47.4%)	0.323
Post-dilation	36/106 (34%)	16/68 (23.5%)	20/38 (52.6%)	0.002
Contrast medium, ml		130.71 ± 47.08	132.76 ± 41.89	0.823
Fluoroscopy, min		14.90 ± 11.85	16.57 ± 9.09	0.454
Dose-length product, mGy × cm^2^		4,557 ± 5,297	5,763 ± 4,146	0.229
Need for permanent pacemaker	17/106 (16%)	8/68 (11.8%)	9/38 (23.7%)	0.109
Procedural death	0/112 (0%)	0/74 (0%)	0/38 (0%)	
Need for second valve	6/106 (5.7%)	3/68 (4.4%)	3/38 (7.9%)	0.457
Annular rupture	1/106 (0.9%)	0/68 (0%)	1/38 (2.6%)	0.358
Coronary obstruction	0/106 (0%)	0/68 (0%)	0/38 (0%)	NA
30-day all-cause death	1/106 (0.9%)	0/68 (0%)	1/38 (2.6%)	0.358
30-day cardiac death	1/106 (0.9%)	0/68 (0%)	1/38 (2.6%)	0.358
Technical success	91/106 (85.8%)	61/68 (89.7%)	30/38 (78.9%)	0.128
Device success (at 30 days)	88/106 (83%)	55/68 (80.9%)	33/38 (86.8%)	0.433
Early safety (at 30 days)	83/106 (78.3%)	57/68 (83.8%)	26/38 (68.4%)	0.065
Patient-prothesis-mismatch				0.059
Moderate	29/106 (27.3%)	23/68 (33.8%)	6/38 (15.8%)	
Severe	9/106 (8.5%)	7/68 (10.3%)	2/38 (5.3%)	
EOA, cm^2^		1.78 ± 0.45	1.85 ± 0.48	0.497
iEOA		0.92 ± 0.23	0.98 ± 0.24	0.145
Mean gradient >20 mmHg predischarge	5/106 (4.7%)	5/68 (7.4%)	0/38 (0%)	0.103
Mean gradient predischarge, mmHg		11.74 ± 5.11	9,01 ± 3.63	0.005
Mean gradient >20 mmHg at 30 days	6/106 (5.7%)	6/68 (8.8%)	0/38 (0%)	0.064
Mean gradient at 30 days, mmHg		11,91 ± 4,58	9,21 ± 3,08	0.002
Aortic regurgitation predischarge echo				0.305
None/minimal	63/106 (59.4%)	44/68 (64.7%)	19/38 (50%)	
Mild	33/106 (31.1%)	19/68 (27.9%)	14/38 (36.8%)	
Mild-moderate	10/106 (9.4%)	5/68 (7.4%)	5/38 (13.2%)	
Moderate	0/106 (0%)	0/68 (0%)	0/38 (0%)	
Moderate-severe	0/106 (0%)	0/68 (0%)	0/38 (0%)	
Severe	0/106 (0%)	0/68 (0%)	0/38 (0%)	
LVEF predischarge, %		53.60 ± 12.37	57.05 ± 10.23	0.151
In-hospital kidney failure				0.572
AKIN 1	3/106 (2.8%)	1/68 (1.5%)	2/38 (5.3%)	
AKIN 2	1/106 (0.9%)	1/68 (1.5%)	0/38 (0%)	
AKIN 3	2/106 (1.9%)	1/68 (1.5%)	1/38 (2.6%)	
AKIN 4	0/106 (0%)	0/68 (0%)	0/38 (0%)	
TIA or stroke	3/106 (2.8%)	2/68 (2.9%)	1/38 (2.6%)	0.547
Vascular complications				0.717
Minor	11/106 (10.4%)	6/68 (8.8%)	5/38 (13.2%)	
Major	4/106 (3.8%)	3/68 (4.4%)	1/38 (2.6%)	
Bleeding				0.301
Type 1	17/106 (16%)	11/68 (16.2%)	6/38 (15.7%)	
Type 2	6/106 (5.7%)	4/68 (5.9%)	2/38 (5.3%)	
Type 3	2/106 (1.9%)	0/68 (0%)	2/38 (5.3%)	
Type 4	0/106 (0%)	0/68 (0%)	0/38 (0%)	

Values are mean ± SD or *n* (%). AKIN, acute kidney injury network; EOA, effective orifice area; iEOA, indexed effective orifice area; LVEF, left ventricular ejection fraction; TIA, transient ischemic attack.

### Statistical analysis

Categorical variables were reported as count and percentage and compared by Pearson's *χ*^2^ or Fisher's exact test as appropriate. Continuous variables distribution was inspected by Shapiro–Wilk test, reported as mean ± standard deviation or median and interquartile range, and compared by *t*-test or Mann–Whitney–*U* test, respectively. All tests were two-sided at the 0.05 significance level. Statistical analysis was completed using IBM SPSS Statistics for Windows, version 25 (IBM Corp., Armonk, NY, USA).

## Results

**Figure 1:**
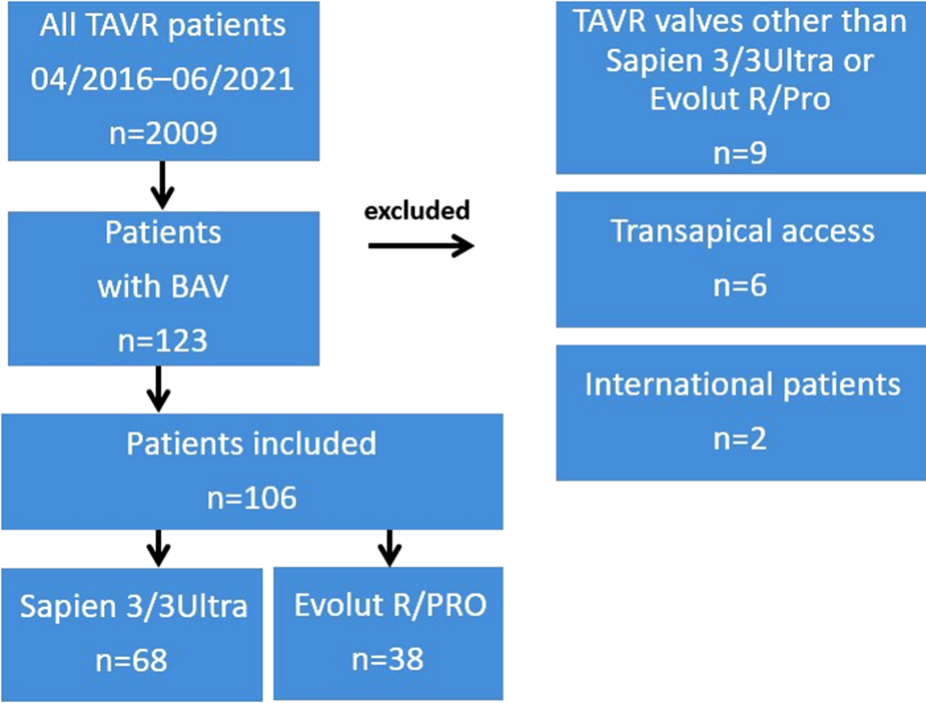
Study design. BAV, bicuspid aortic valve; TAVR, transcatheter aortic valve replacement.

One hundred and six patients with bicuspid aortic valves who underwent transcatheter aortic valve implantation using BEV (*n* = 68) or SEV (*n* = 38) prostheses were included in our study (Figure 1).

### Baseline characteristics

There were significantly more men in the BEV group (72.1% vs. 52.6%; *p* = 0.044), with higher rates of coronary artery disease (48.5% vs. 31.6%; *p* = 0.090) and cerebrovascular disease (7.4% vs. 0%, *p* = 0.083). Surgical risk scores were comparable in both groups (mean STS PROM 2.6 ± 1.9% vs. 2.6 ± 1.6%; *p* = 0.374, mean EuroSCORE II 3.9 ± 3.3 vs. 2.8 ± 1.6; *p* = 0.074, and mean EuroSCORE log 11.6 ± 9.2 vs. 9.3 ± 6.3; *p* = 0.165, in BEV vs. SEV, respectively). Baseline characteristics are shown in Table 1.

**Figure 2:**
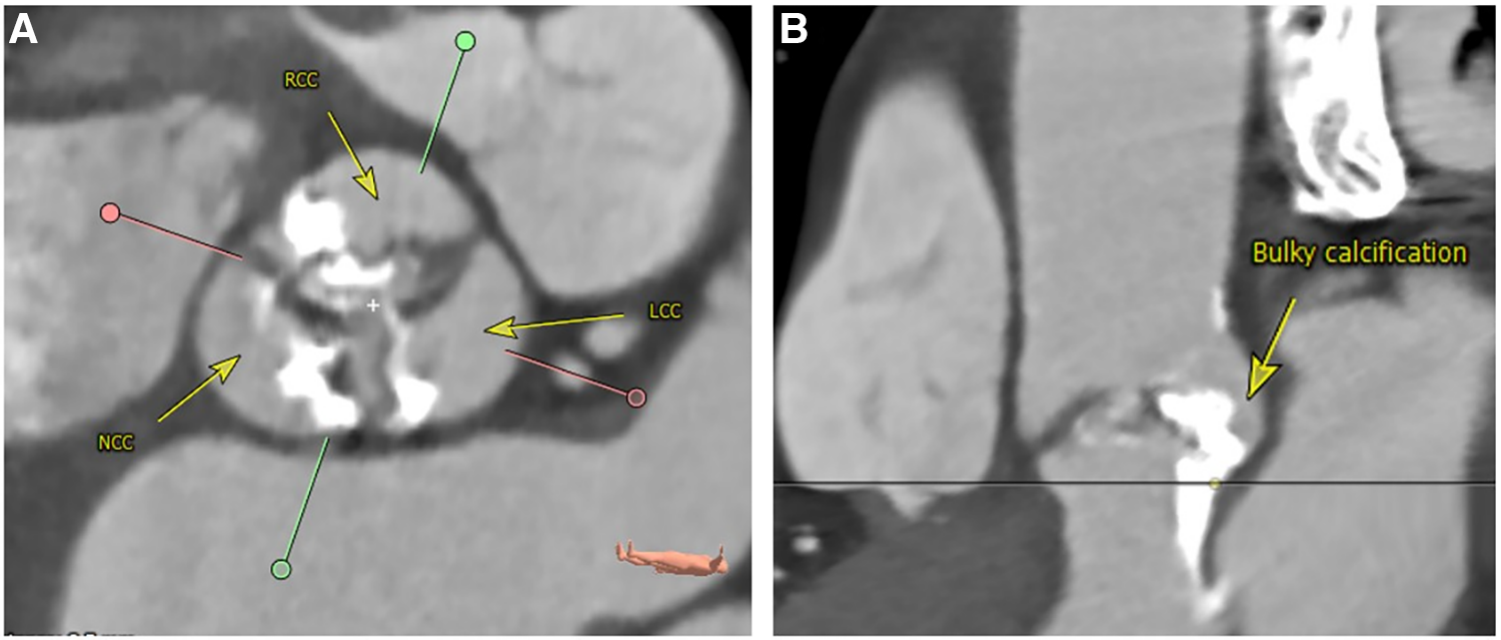
Bulky asymmetrical calcification of the aortic valve. Perpendicular plane (**A**): sievers type I L/R bicuspid valve with heavy calcification. Stretched vessel view (**B**): bulky and asymmetrical calcification ranging from the aortic valve and annulus into the LVOT underneath the area between NCC and LCC. LCC, left coronary cusp; LVOT, left ventricular outflow tract; L/R, left/right; NCC, non-coronary cusp; RCC, right coronary cusp. Reconstructions from computed angiotomography using 3Mensio software, pie medical imaging BV, Maastricht, The Netherlands.

The majority of BAV were bicommissural raphe type anatomy (Sievers Type 1) with fusion of the left and right coronary cusps (overall 82.1%, BEV 83.8% vs. SEV 78.9%; *p* = 0.119). Regarding the volume of calcification of the aortic valve, there was no significant difference in the two groups [DLZ-CV (mm^3^) BEV 1,168 ± 811 vs. SEV 948 ± 850, *p* = 0.192]. Qualitative assessment of aortic valve calcification (none, mild, moderate, severe) showed that moderate calcification was most common without significant difference between the two groups (BEV 54.4% vs. SEV 55.3%; *p* = 0.445). A bulky asymmetrical calcification pattern was identified in BEV 32.4% vs. SEV 28.9%; *p* = 0.446 (Figure 2).

### Procedural characteristics and in-hospital outcomes

**Figure 3:**
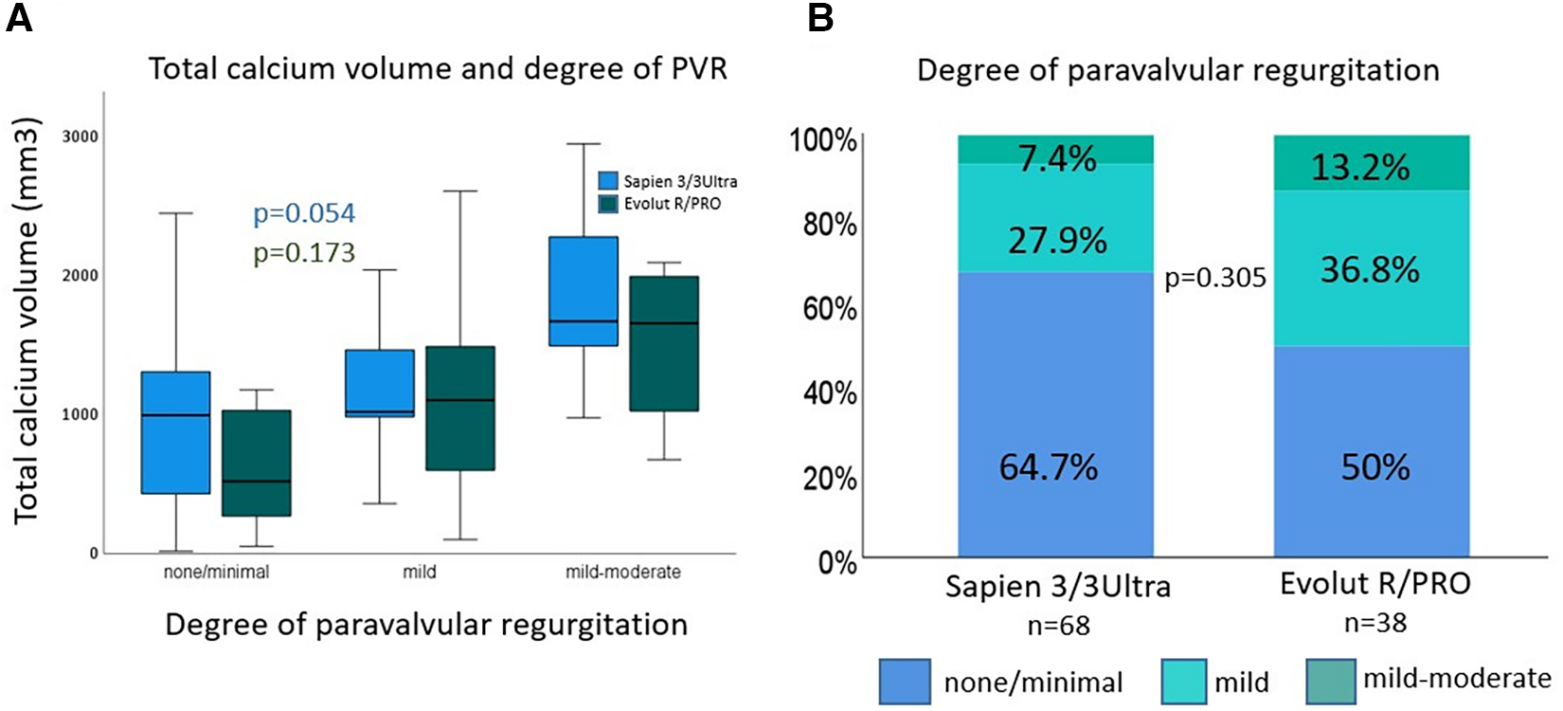
Device landing zone calcium volume (DLZ-CV) and degree of paravalvular regurgitation. Device landing zone calcium volume and degree of paravalvular regurgitation (**A**): DLZ-CV had an impact on degree of PVL, which was significant for Sapien 3/3 Ultra devices. Degree of paravalvular regurgitation (**B**): percent of degree of paravalvular regurgitation at 30 days follow-up was reduced with Sapien 3/3 Ultra devices. DLZ-CV, device landing zone calcium volume; PVL, paravalvular regurgitation.

Procedural characteristics and clinical outcomes are shown in Table 2. Pre-dilatation was performed in 57.4% vs. 47.4% of cases, in BEV vs. SEV, respectively (*p* = 0.323). In the SEV group post-dilatation was performed significantly more often (52.6% vs. 23.5%; *p* = 0.002). Predominantly non-compliant balloons were used for post-dilation. For post-dilation of BEV the balloon which is supplied with the delivery catheter was used. There was no procedural mortality. One annulus rupture requiring emergency surgery occurred in the SEV group (2.6% vs. 0.0%; *p* = 0.358). There was no significant difference in the two groups regarding the frequency of procedural coronary obstruction (none), bleeding type 3 and 4 (none), and major vascular complications (BEV 4.4% vs. SEV 2.6%; *p* = 0.717). Acute kidney injury network grade 3 (AKIN 3) was 1.5% vs. 2.6% (*p* = 0.572) in BEV vs. SEV, respectively, and none of the patients required renal replacement therapy (AKIN 4). Need for a second THV was similar in the SEV and BEV group (7.9% vs. 4.4%; *p* = 0.457). Mild-moderate postoperative paravalvular regurgitation occurred in 7.4% in the BEV group vs. 13.2% in the SEV group (*p* = 0.305). Paravalvular regurgitation > mild-moderate was not observed. DLZ-CV had an influence on the degree of postoperative aortic regurgitation regardless of valve prosthesis design and reached significance for BEVs (*p* = 0.054; Figure 3). VARC-3 “technical success” rate did not differ significantly between the groups (BEV 89.7% vs. SEV 78.9%; *p* = 0.128).

### Thirty-day outcomes

There were no significant differences in the components of the VARC-3-“early safety” endpoint (see Table 2). Specifically, no deaths occurred in the BEV group and 1 death in the SEV group (2.6% vs. 0.0%; *p* = 0.358). The TIA/stroke rate in both groups was comparable (BEV 2.9% vs. SEV 2.6%; *p* = 0.547). Valve Academic Research Consortium-3 (VARC-3) device success at 30 days was similar between BEV and SEV (80.9% vs. 86.8%; *p* = 0.433). Mean gradient >20 mmHg at 30 days occurred more often in the BEV group (8.8%vs. 0.0%; *p* = 0.064). Mean gradient (mmHg) at 30 days was 11.91 ± 4.58 in the BEV group vs. 9.21 ± 3.08 in the SEV group (*p* = 0.002). There was a trend towards increased need for permanent pacemaker implantation in the SEV group (23.7% vs. 11.8%; *p* = 0.109).

## Discussion

This single-center, retrospective study compared 106 consecutive patients undergoing TAVR with the latest generation of the two most commonly used THVs (SAPIEN 3/3Ultra vs. Evolut R/PRO) in BAV anatomy. Current TAVR practice is mainly based on evidence on TAVR for tricuspid AV (TAV), as BAV anatomy has been an exclusion criterion in the large landmark TAVR trials (11–15). With younger and lower-risk patients undergoing TAVR in the future, the frequency of BAV anatomy is likely to increase. Therefore, it is essential to optimize TAVR outcomes in this particular patient subset (20). To our knowledge this is the first study using the VARC-3 criteria for standardized endpoint reporting for TAVR in BAV anatomy.

The main findings of our study are as follows:

1. Valve Academic Research Consortium-3 (VARC-3) device success at 30 days did not differ between BEV and SEV.
2. SEV and BEV displayed similar results on paravalvular aortic regurgitation with none exceeding mild-moderate regurgitation.
3. In the SEV group, post-dilatations were performed significantly more often, not resulting in significant differences regarding annular rupture and TIA/Stroke.
4. Increasing DLZ-CV was associated with higher degree of paravalvular regurgitation.
5. SEV showed significantly lower transvalvular gradients at 30-days follow-up.

### Device success at 30 days

Pearlman et al. reported an excellent VARC-2 device success rate of 98% using the Sapien 3 THV in 51 patients with BAV anatomy (7). Encouraging results of 929 patients have been published from the Society of Thoracic Surgeons (STS)/American College of Cardiology (ACC) Transcatheter Valve Therapies (TVT) Registry with the use of the Evolut R/PRO platform in BAV anatomy reporting a device success rate of 96.5% (19). Mangieri et al. published data from a multi-center registry [balloon vs. self-expandable valve for the treatment of bicuspid aortic valve stenosis (BEAT) registry] including 353 consecutive patients and a VARC-2 device success rate of 85.7% vs. 84.4% with SAPIEN 3 vs. Evolut R/PRO, respectively (*p* = 0.821) (30). According to the up-to-date VARC-3 criteria we achieved 80.9% vs. 86.8% 30-day device success for BEV and SEV in BAV anatomy, respectively (*p* = 0.433).

### Paravalvular leakage

In BAV anatomy, low (0% to 2.5%) rates of PVL ≥ moderate were reported for Sapien 3 (7, 22, 31). Forrest and colleagues reported results from the STS/ACC TVT Registry with the use of the Evolut R/PRO THV in BAV anatomy (*n* = 929) describing post-procedural > mild PVL of 5.6% (19). For the Evolut R/PRO THV no case of PVL ≥ mild was reported in 150 individuals included into a prospective study, also by Forrest et al., on low risk BAV patients at 30 days (20). A retrospective multicenter study by Mylotte et al. demonstrated trend towards increased rates of post-implantation aortic regurgitation ≥ mild using earlier-generation SEVs (SapienXT 19.6% vs. CoreValve 32.2%, *p* = 0.11) (16). Mangieri and co-workers found that in 77 matched patients pairs with BAV (BEAT registry), ≥moderate-severe PVL was 10.4% with Evolut R/PRO compared to 0% with Sapien 3 (*p* < 0.004) (30). The results of the present study compare favourably with previous studies with none of the patients displaying > mild-moderate PVL and comparable mild-moderate PVL rates among SEV and BEV.

### Post-dilatation, need for new pacemaker, stroke, annular rupture, and need for second valve

Mylotte and colleagues found a trend towards more post-dilations in earlier-generation SEVs vs. BEVs (CoreValve 22.2% vs. SapienXT 10.6%; *p* = 0.11) without an influence on PPI (SEV 26.7% vs. BEV 16.7%; *p* = 0.21) and stroke rate (SEV 2.2% vs. BEV 2.1%; *p* = 0.99) (16). Accordingly, Mangieri et al. reported significantly more post-dilations in SEV (42.7%) compared to BEV 14.3% (*p* < 0.001), without impact on need for PPI (SEV 14.3% vs. BEV 17.1%, *p* = 0.642) or stroke (SEV 1.5% vs. 0%; *p* = 0.323) (30). In the present study post-dilations were performed in 52.6% of Evolut R/PRO vs. 23.5% of Sapien 3/3 Ultra (*p* = 0.002). Moreover, the need for new permanent pacemaker implantations trended to be more frequent after TAVR with SEV without reaching statistical significance. The 30-day stroke rate was comparable between SEV and BEV (2.6% vs. 2.9%; *p* = 0.547) in the present study. Consistent with our findings, a recent meta-analysis of BEV vs. SEV in BAV anatomy using pooled odds ratio and conclusions plot across five studies using the Mantel–Haenszel method did not report a significant difference in the incidence of stroke at 30 days (32).

Makkar et al. reported data from the STS/ACC TVT Registry from low-risk patients (STS risk score 1.7 ± 0.7) having undergone TAVR using Sapien 3/3 Ultra in BAV anatomy (*n* = 3,168) showing an annular rupture rate of 0.2% and need for second valve rate of 0.3% (33). Newer generation THV displayed a comparable incidence of annular rupture of 1.7% with BEV and none with SEV in BAV anatomy (*p* = 0.173) in a multicenter registry of consecutive BAV stenosis undergoing TAVR (BEAT registry) (30). In our study one patient with Sievers type 1 L/R and severe calcification of the aortic valve (DLZ-CV 1,143 mm^3^) suffered from an annular rupture following implantation of an Evolut PRO THV with post-dilation requiring emergency surgery. We were able to show a trend towards increased need for second valve implantation with SEV (7.9% vs. 4.4%; *p* = 0.457). Similar findings are reported from the BEAT registry indicating a trend towards more frequent need for second valve with SEV in a matched population (SEV 6.5% vs. BEV 1.3%; *p* = 0.096) (30).

### Calcium volume and paravalvular leakage

The association of DLZ-CV with PVL is currently in the focus of intense research. Previous reports showed that aortic valve calcium was predictive of a higher PVL rate for early-generation SEV (SAPIEN XT vs. CoreValve) (34, 35). Pollari and co-workers retrospectively analyzed preoperative contrast-enhanced MDCT scans of patients who underwent TAVR in a single-center cohort using various THVs including Sapien 3 (*n* = 206) and CoreValve/Evolut R (*n* = 44). Using a logistic regression model they demonstrated that DLZ-CV [OR 1.08; 95% CI (1.04–1.12); *p* = 0.00006] and use of CoreValve/Evolut R prosthesis [OR 4.09; 95% CI (1.62–10.3); *p* = 0.003] were associated with mild or greater PVL. Conversely, the use of Sapien 3 was associated with a lower incidence of mild or greater PVL [OR 0.23; 95% CI (0.11–0.47); *p* = 0.00005] (36). Kim et al. showed that the procedural outcome of TAVR using BEVs was independent of the DLZ-CV, whereas DLZ-CV was significantly higher in patients with PVL ≥1° or those requiring post-dilatation of SEVs (SAPIEN 3 vs. Acurate Neo) (24). Moreover, a single-center study comparing results of TAVR with Sapien 3 in bicuspid and tricuspid aortic valves demonstrated that the volume of calcification was significantly greater in BAV anatomy (1,272 mm^3^ vs. 803 mm^3^; *p *< 0.001) (37). Watanabe and co-workers published similar results following TAVR in bicuspid and tricuspid aortic valves (BAV DLZ-CV 1,262.76 ± 396.0 mm^3^ vs. TAV DLZ-CV 556.46 ± 461.9 mm^3^; *p* < 0.01) (38). In the present study DLZ-CV was associated with the degree of postoperative aortic regurgitation for both groups, however, only reaching significance for BEVs (*p* = 0.054). Albeit, none of the patients displayed ≥ moderate PVL and had comparable mild-moderate PVL rates in both groups.

### Hemodynamic performance

Large registry data of patients with BAV anatomy and BEV (Sapien 3/3Ultra, unmatched population *n* = 6,995) published by Makkar et al. detected a mean gradient >20 mmHg 7.4% at discharge and mean gradient 12.3 ± 5.4 mmHg at 30-days follow-up (33). Forrest et al. reported post-procedural hemodynamic results from the STS/ACC TVT Registry for SEV (Evolut R/PRO, *n* = 929): mean gradient 9.7 ± 5.2 mmHg and 5.9% mean gradient >20 mmHg (19). In another study by Forrest and co-workers the mean gradient was 7.6 ± 3.7 mmHg and mean gradient >20 mmHg was 1.4% at 30-days follow-up (20). Mangieri et al. reported comparable in-hospital hemodynamic outcomes with a lower incidence of mean gradient >20 mmHg (SEV 5.2% vs. BEV 9.1%; *p* = 0.348) and significantly lower mean gradient of SEV 9.7 ± 4.9 mmHg vs. BEV 11.5 ± 4.3 mmHg (*p* = 0.026) in patients with BAV anatomy (matched populations BEAT registry, *n* = 154) (30). In accordance, in the present study at 30-days follow-up the mean gradient was significantly lower for SEV vs. BEV (9.21 ± 3.08 mmHg vs. 11.91 ± 4.58 mmHg; *p* = 0.002) whereas the proportion of patients with mean AV gradient >20 mmHg was higher in the BEV group without reaching statistical significance (8.8% vs. 0%, *p* = 0.064). The superior hemodynamic properties of SEVs might be explained with the supra-annular design providing a larger (indexed) effective orifice area for these prostheses.

### Future perspectives

In the light of previously published (BEAT) and our own data, a randomized controlled trial comparing SEV and BEV in BAV might be helpful.

### Study limitations

This study carries the inherent limitations of an observational study without independent adjunction of adverse events and without an independent core laboratory. There was an unbalanced aortic dimension in the BEV and SEV group potentially biasing the outcome parameters. A potential impact of unknown or unmeasured confounding factors on study outcomes cannot be excluded.

## Conclusions

Our study confirms the valid use of both, BEV and SEV in bicuspid aortic valve anatomy. VARC-3 device success at 30 days was similar between BEV and SEV, with self-expandable THVs displaying lower transvalvular gradients. On the other hand, there was a trend towards more PVL in the SEV group. From the present study no clear recommendation for SEV or BEV can be given for a preferred selection of BEV or SEV in bicuspid aortic valve anatomy.

## Data Availability

The raw data supporting the conclusions of this article will be made available by the authors, without undue reservation.
